# McCune Albright syndrome is a genetic predisposition to intraductal papillary and mucinous neoplasms of the pancreas associated pancreatic cancer in relation with *GNAS* somatic mutation – a case report

**DOI:** 10.1097/MD.0000000000018102

**Published:** 2019-12-16

**Authors:** Sébastien Gaujoux, Eric Pasmant, Caroline Silve, Nadia Mehsen-Cetre, Romain Coriat, Alexandre Rouquette, Bertrand Douset, Frédéric Prat, Karen Leroy

**Affiliations:** a Department of Digestive, Hepato-biliary and Pancreatic Surgery, Cochin Hospital, APHP; b Faculté de Médecine Paris Descartes, Université Paris Descartes, Sorbonne Paris Cité; c INSERM Unité 1016, Centre National de la Recherche Scientifique Unité Mixte de Recherche 8104, Institut Cochin; d Service de Génétique et Biologie Moléculaires, Hôpital Cochin; e EA7331, Université Paris Descartes; f Department of Gastroenterology, Cochin Hospital, APHP; g Service de Rhumatologie CHU Bordeaux-Tripode; h INSERM U1169, Hôpital Bicêtre; i Centre de Référence des Maladies Rares du Métabolisme du Calcium et du Phosphore / Filière OSCAR; j Department of Pathology, Cochin Hospital, APHP, Paris, France.

**Keywords:** *GNAS*, IPMN, McCune Albright syndrome, secretin-test

## Abstract

**Rationale:**

Intraductal papillary and mucinous neoplasms of the pancreas (IPMN) are preneoplastic lesions diagnosed with an increasing incidence. Recently, several groups have described, in up to 70% of IPMN, activating mutations of the G-protein alpha stimulatory sub-unit (Gsα subunit) gene (*GNAS*). *GNAS*-activating somatic, post-zygotic, mutations are also associated with McCune-Albright syndrome (MCAS) characterized by fibrous dysplasia, precocious puberty, and café-au-lait spots.

**Patient concerns:**

We herein report a patient with McCune Albright Syndrome that presented with malignant IPMN and underwent pancreatic resection.

**Diagnoses and interventions:**

Leucocyte and duodenum juice DNA analysis, endoscopically collected from secretin-stimulated pancreatic juice revealed the same (*GNAS*) activating mutation also found in the invasive pancreatic colloid adenocarcinoma arising from intestinal subtype IPMN.

**Outcomes:**

Thirty months after surgery, the patient was alive with recurrence (bone only metastasis).

**Lessons:**

In this observation, we show that MCAS should be view as a new genetic predisposition to IPMN associated pancreatic cancer, and consequently a targeted screening in this high-risk population might be proposed.

## Abstract 2

Supplemental Digital Content is available in the text

## 1 Introduction

McCune-Albright syndrome (MCAS) is a rare disorder characterized by polycystic fibrous dysplasia, precocious puberty, and *café au lait* spots. It is caused by somatic, post-zygotic, with mosaic distribution, *GNAS* activating mutations.^[1]^ In addition to other hepatobiliary neoplasms,^[2,3]^ IPMN have been associated with MCAS.

Somatic activating mutations of the G-protein alpha stimulatory subunit (Gsα subunit) encoded by the *GNAS* gene (*GNAS*) have been reported in up to 70% of pancreatic intraductal papillary mucinous neoplasms (IPMN),^[4–7]^ that is a precursor of pancreatic adenocarcinoma. In this setting, *GNAS* mutations, known to lead to elevated intracellular cAMP levels and activation of downstream dependent pathways,^[1]^ open new clinical insights on IPMN. As an example, IPMN intestinal pattern of differentiation is associated with *GNAS* mutation^[8]^ underlining the functional consequences of *GNAS* activating mutation. Once symptomatic, pancreatic adenocarcinoma is associated with a dismal prognosis. Identifying individuals at risk and detecting early lesions are crucial to improve patient's outcome. Several conditions have been found to be associated with an increased risk of pancreatic adenocarcinoma, and targeted screening of high-risk individuals is important.

The aim of the present study is to examine the mutation status of *GNAS* in a patient with McCune Albright Syndrome and IPMN who underwent pancreatic resection.

## 2 Case report

**Figure 1:**
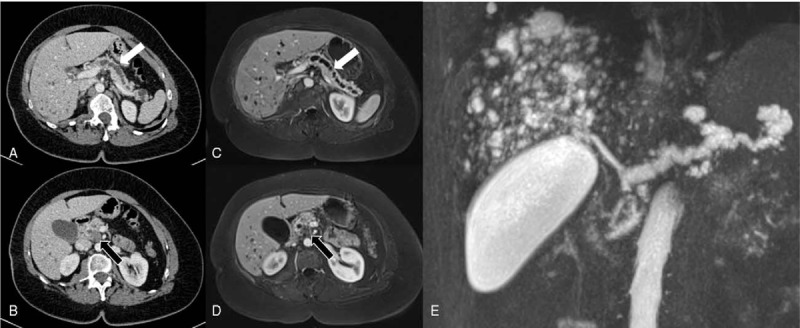
Cross sectional imaging. A: Portal phase enhanced CT scan showing main pancreatic duct dilatation (white arrow). B: Portal phase enhanced CT scan showing pancreatic head adenocarcinoma (black arrow). C: T1 Gadolinium enhanced MRI showing main pancreatic duct dilatation (white arrow). D: Gadolinium enhanced MRI showing pancreatic head adenocarcinoma (black arrow). E: CP-MRI showing main pancreatic duct dilatation.

**Figure 2:**
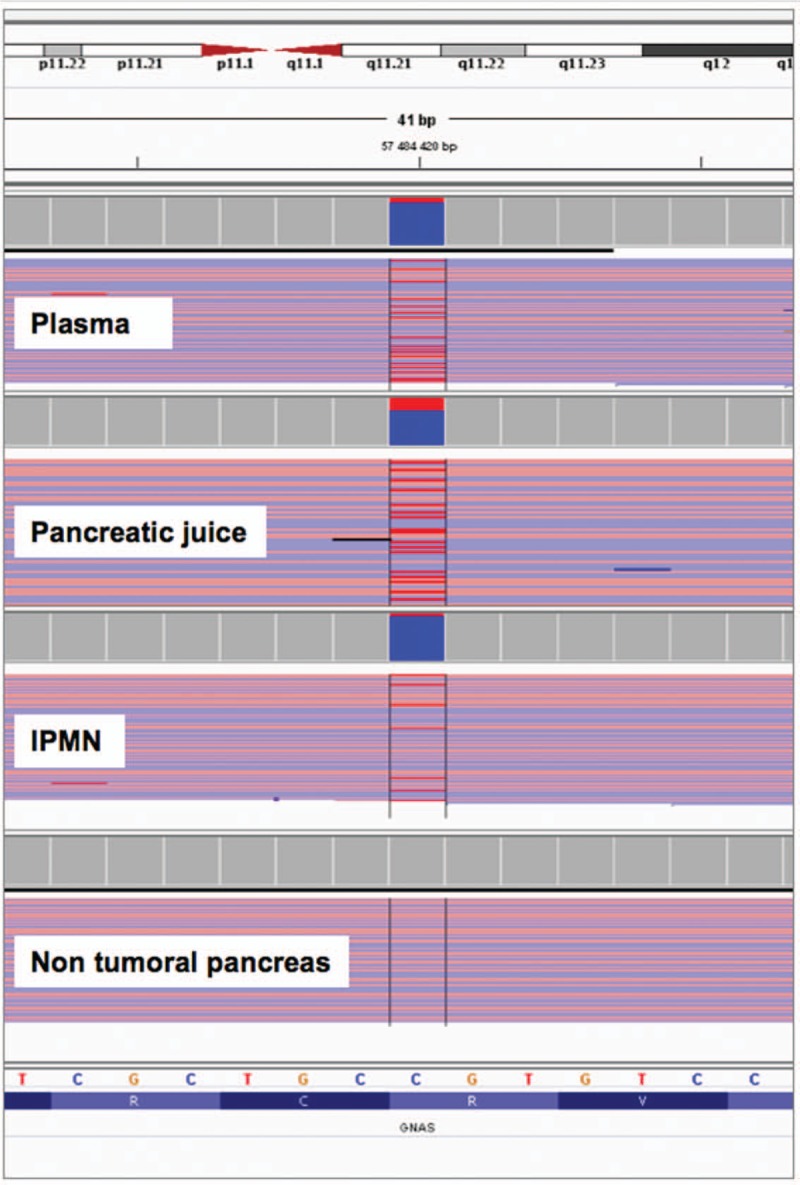
*GNAS* sequencing reads: *GNAS* (NM_000516): c.601C>T, p.Arg201Cys mutation was detected in 13% of NGS reads in plasma, 29% sequencing reads in pancreatic juice, 8% sequencing reads in IPMN associated cancer and was not detected in non-tumoral pancreas (threshold set at 2%). Reads were visualized by uploading bam files into Integrative Genomics Viewer the variant T allele is shown in red.

A 50-year old woman, 144 cm for 58 kg, initially presented with abdominal pain. Patient has provided informed consent for publication of the case. She was previously diagnosed with McCune Albright Syndrome (MCAS) with severe fibrous dysplasia and precocious puberty. She had a past medical history of total thyroidectomy, and multiple surgery for fractures. Cross sectional imaging (Fig. 1) and endoscopic ultrasonography reveled a global main pancreatic duct dilatation over 10 mm associated with a cephalic 25-mm enhanced mural nodule with portal vein lateral abutment, without distant metastasis. Fine-needle aspiration pathology confirmed an IPMN related colloid pancreatic adenocarcinoma. Leucocyte and duodenum juice deoxyribose nucleic acid (DNA) analysis^[9]^ (endoscopically collected from secretin-stimulated pancreatic juice; Supplemental Video) revealed the same G-protein alpha stimulatory sub-unit (Gsα subunit) gene (*GNAS*) (NM_000516) activating mutation c.601C>T (p.Arg201Cys) (Fig. 2). The same mutation was also detected in plasma circulating DNA. The patient underwent pancreaticoduodenectomy after 3 months of FOLFIRINOX neoadjuvant chemotherapy. Pathological examination revealed an invasive pancreatic colloid adenocarcinoma (Supplemental Fig.) (ypT2 N1 R0) arising from intestinal subtype IPMN (ie, MUC1-, MUC2+, MUC5AC+ immunohistochemistry) with a major (over 90%) response to chemotherapy. Genetic analysis of the IPMN revealed a *GNAS* (NM_000516) activating mutation c.601C>T (p.Arg201Cys), which was not detected at a 2% variant allele frequency threshold in the adjacent normal pancreas. No *KRAS* or other driver mutation was detected in the IPMN associated cancer with a targeted 50 genes NGS panel. Imaging and medical work-up, plasma circulating tumor DNA, Formalin-Fixed Paraffin Embedded and pancreatic juice somatic mutation analysis technics are available in Supplemental Material. Thirty months after surgery, the patient is alive with recurrence (bone only metastasis).

## 3 Discussion

Intraductal papillary mucinous neoplasm (IPMN) can be seen as a “recent” disease and has only been clearly individualized in the mid 80's. Since then, its diagnosis, description, comprehension and management have been significantly improved.^[10–13]^

The adenoma-carcinoma sequence leading to the development of pancreatic adenocarcinoma is currently under investigation but include as major events telomere shortening, *KRAS* activating mutation, loss and/or mutation of *SMAD4* and *p53*.^[14]^ Since recent whole-exome analysis, *GNAS* mutations also appear to have a key role in IPMN pathogenesis.^[15]^ With *KRAS*, it is therefore, one of the 2 most prevalent mutations in these tumors. It may occur alone or in association with *KRAS* activating mutations and could define a specific progression pathways in IPMN-associated carcinoma.^[15–17]^*GNAS*-activating mutations are reported in both IPMN and MCAS, and IPMN is a MCAS associated lesion.^[18,19]^ This emphasizes the important role of *GNAS* in pancreatic tumorigenesis.^[5]^*GNAS*- driven pancreatic tumorigenesis is associated with IPMN intestinal phenotype^[8]^ and colloid pancreatic adenocarcinoma,^[16]^ and a less aggressive disease, with better long-term outcome.^[20]^ IPMN occur most of the time as a sporadic disease. Some^[21–23]^ previously reported familial forms of IPMN in few kindred, suggesting predisposing genetic alteration. So far they were not found, and neither *BRCA2*, *p16* nor *CDKN2A* were constitutionally mutated or lost.^[21]^ If familial forms of pancreatic adenocarcinoma are now well known,^[24,25]^ familial forms of pancreatic adenocarcinoma have not been formally described.

First, this observation underlines the need for a specific screening for high-risk patients identified by their known genetic predisposition, and MCAS should be considered as a pancreatic cancer predisposition syndrome. Second, it is now possible to determine preoperatively *GNAS* status from plasma circulating DNA, duodenum juice DNA collected after secreting stimulation test, or DNA from extracted paraffin-embedded tissue from EUS-FNA and to identify at least intestinal IPMN phenotype. If up to now this information has a limited value,^[26]^ it is likely that in a near future it will help to tailor pancreatic cyst and IPMN management.^[27]^

Overall, this observation provides additional evidence of MCAS as a new genetic predisposition to IPMN associated pancreatic cancer, and consequently the need for a specific screening in this population.

## Author contributions

**Conceptualization:** Sebastien Gaujoux, Eric Pasmant, Caroline Silve, Frédéric Prat, Karen Leroy.

**Data curation:** Sebastien Gaujoux, Romain Coriat, Frédéric Prat, Karen Leroy.

**Formal analysis:** Sebastien Gaujoux, Eric Pasmant, Caroline Silve, Nadia Mehsen-Cetre, Alexandre Rouquette, Bertrand Dousset, Frédéric Prat, Karen Leroy.

**Funding acquisition:** Sebastien Gaujoux, Romain Coriat, Bertrand Dousset, Frédéric Prat, Karen Leroy.

**Investigation:** Sebastien Gaujoux, Caroline Silve, Nadia Mehsen-Cetre, Bertrand Dousset, Frédéric Prat, Karen Leroy.

**Methodology:** Sebastien Gaujoux, Eric Pasmant, Caroline Silve, Romain Coriat, Alexandre Rouquette, Frédéric Prat, Karen Leroy.

**Project administration:** Sebastien Gaujoux, Eric Pasmant, Romain Coriat, Alexandre Rouquette, Frédéric Prat, Karen Leroy.

**Resources:** Sebastien Gaujoux, Caroline Silve, Nadia Mehsen-Cetre, Bertrand Dousset, Frédéric Prat, Karen Leroy.

**Software:** Sebastien Gaujoux, Eric Pasmant, Alexandre Rouquette, Frédéric Prat, Karen Leroy.

**Supervision:** Sebastien Gaujoux, Caroline Silve, Romain Coriat, Alexandre Rouquette, Frédéric Prat, Karen Leroy.

**Validation:** Sebastien Gaujoux, Nadia Mehsen-Cetre, Romain Coriat, Bertrand Dousset, Frédéric Prat, Karen Leroy.

**Visualization:** Sebastien Gaujoux, Eric Pasmant, Alexandre Rouquette, Frédéric Prat, Karen Leroy.

**Writing – original draft:** Sebastien Gaujoux, Frédéric Prat, Karen Leroy.

**Writing – review & editing:** Sebastien Gaujoux, Eric Pasmant, Caroline Silve, Nadia Mehsen-Cetre, Romain Coriat, Alexandre Rouquette, Bertrand Dousset, Frédéric Prat, Karen Leroy.

Sebastien Gaujoux orcid: 0000-0002-1072-7639.

## Supplementary Material

Supplemental Digital Content

## Supplementary Material

Supplemental Digital Content

## Supplementary Material

Supplemental Digital Content
