# Supplementary material for: McCune Albright syndrome is a genetic predisposition to intraductal papillary and mucinous neoplasms of the pancreas associated pancreatic cancer in relation with GNAS somatic mutation – a case report
Source: Medicine (Baltimore). 2019 Dec 16;98(50):e18102. doi: 10.1097/MD.0000000000018102 (PMC6922479; doi:10.1097/MD.0000000000018102)
Supplement: Supplemental Digital Content [file medi-98-e18102-s002.doc]

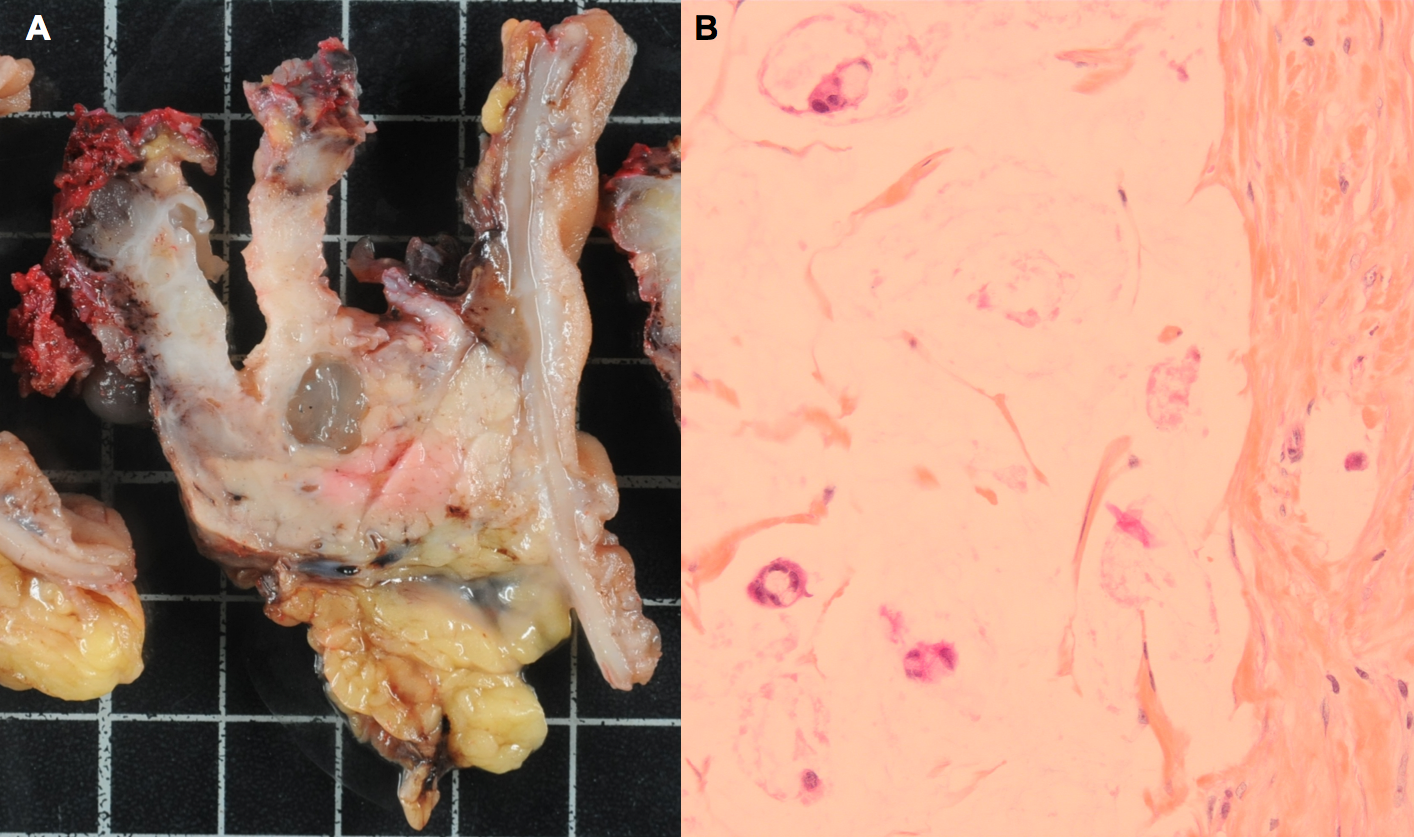


**Supplemental Figure:**

A: Gross aspect of the specimen after pancreaticoduodenectomy

B: HES staining of colloid pancreatic adenocarcinoma with stromal pools of acellular mucin with floating tumor cells (X20)
