# Supplementary material for: McCune Albright syndrome is a genetic predisposition to intraductal papillary and mucinous neoplasms of the pancreas associated pancreatic cancer in relation with GNAS somatic mutation – a case report
Source: Medicine (Baltimore). 2019 Dec 16;98(50):e18102. doi: 10.1097/MD.0000000000018102 (PMC6922479; doi:10.1097/MD.0000000000018102)
Supplement: Supplemental Digital Content [file medi-98-e18102-s003.docx]

*Imaging and medical work-up*

Permission from Institutional Review Board (AAA-2017-05005) was obtained prior data review and analysis. Clinical work-up included physical examination, dual-phase thin-section multidetector CT, hepatic and pancreatic enhanced magnetic resonance imaging with diffusion weighted imaging, endoscopic ultrasonography with guided fine-needle aspiration, and pancreatic juice sampling collected from the duodenum 5 minutes after stimulation by infusing human synthetic secretin-test (0.2 mg/kg intravenously over a minute).

*Plasma Circulating Tumor DNA, Formalin-Fixed Paraffin Embedded and pancreatic juice somatic mutation analysis*

Blood samples (9 mL) were collected in Cell free DNA BCT tubes (Streck). The samples were centrifuged at 1600g for 10 minutes and a second time at 16000g for 10 minutes before storage at -20°C until extraction. DNA was extracted from plasma and duodenal juice with QIAamp Circulating Nucleic Acid Kit (Qiagen) according to the manufacturer's instructions. DNA was extracted from formalin fixed and paraffin embedded (FFPE) tumor specimens using the Maxwell 16 FFPE Plus LEV DNA Purification (Promega) according to the manufacturer instructions. DNA quantity was assessed using the Qubit dsDNA HS (high sensitivity) Assay Kit (Thermo Fisher). Sequencing libraries were prepared from DNA using Ion AmpliSeq Cancer Hotspot Panel v2 (Thermo Fisher), screening about 2,800 COSMIC mutations of 50 oncogenes and tumor suppressor genes. 4 to 10 ng DNA were used as input for library preparation. The pooled barcoded libraries were processed on Ion Chef System using the Ion PGM Hi-Q Chef Kit (code-barres IonDX) and sequenced on the Ion PGM system and analysed using the Torrent Suites 4.4 and the Ion reporter 5.2. SNV and indel detection threhold was set at 2% and results were given for a minimum of 300X read depth.
